# Special Issue: The Diagnosis and Management of OSA

**DOI:** 10.3390/diagnostics12081919

**Published:** 2022-08-08

**Authors:** Bilgay Izci Balserak

**Affiliations:** Biobehavioral Nursing Science, Center for Sleep and Health Research, College of Nursing, University of Illinois, 845 South Damen Avenue (MC 802), Chicago, IL 60612-7350, USA; bilgay@uic.edu

Obstructive sleep apnea (OSA) is the most common form of sleep-disordered breathing (SDB) and is demonstrating an increasing prevalence worldwide. Its impact on health is significant and serious. Thus, the efficient screening, diagnosis, and treatment of OSA is important for adults as well as for children to prevent the adverse health outcomes associated with OSA. The articles published in this Special Issue highlight these issues. 

The study by Dr. Bublitz and her coworkers in a pregnant population revealed that subclinical levels of SDB in early pregnancy measured by an in-home sleep study, but not those measured by self-reported SDB, predicted elevated depressive symptoms in late pregnancy. Nevertheless, SDB in late pregnancy was not associated with depressive symptoms [1]. These findings suggest that SDB may lead to an increased risk of depressive symptoms during pregnancy.

The following articles focused on screening tools using different participant characteristics, including anthropometric and electroencephalogram (EEG) signals to identify individuals with OSA and to determine its severity. A systematic review and meta-analysis describing the performance of prediction models for OSA during pregnancy conducted by Drs. Siriyotha and Tantrakul et al. provided evidence that BMI, age, and snoring were the most common predictors of OSA among the strong prediction models. When combining BMI and age as continuous variables, the models consistently showed good results [2]. The study by Dr. Hsieh and et al. provides a practical perspective for clinicians to predict severe pediatric OSA more efficiently [3]. Their results suggest that home sleep pulse oximetry combined with the adenoidal–nasopharyngeal ratio (ANR) can screen for severe OSA better than ANR and tonsil size among children with habitual snoring. Drs. Elwali and Moussavi presented the results of their study, which showed the possibility of predicting AHI and other OSA severity parameters with high precision during wakefulness using only anthropometric information and tracheal breathing sounds [4]. Thus, their proposed methodology has the potential to be a quick and reliable OSA screening tool for adults. A study by Drs. Jayaraj and Mohan investigated the sub-band decomposition of EEG signals to identify healthy individuals versus patients with OSA and obtained improved results using a support vector machine classifier [5]. 

The next four articles pertain to the management of sleep apnea and indicate that clinicians and researchers should consider the differences in social and environmental factors when assessing the responses to PAP therapy in patients from different patient groups, determining the need for adjunctive treatments, and designing adherence promotion interventions. Importantly, policymakers should consider these differences when setting patient-centered PAP adherence goals rather than using a one-size-fits-all threshold. The article written by Dr. Imamyama and her coworkers indicates that Black populations have a different response to positive airway pressure (PAP) treatment in terms of sleep outcomes, including wakefulness after sleep onset and the frequency of awakening during the night. In moderate to severe OSA, PAP adherence promotion in Black populations is crucial, and race-specific PAP adherence thresholds need to be considered for clinically meaningful improvements in functional and sleep outcomes [6]. This will help bridge the racial disparities in OSA treatment and outcomes. Dr. Zota and her coworker assessed the impact of short-term CPAP on ventricular function in patients with moderate–severe OSA and cardiometabolic comorbidities. Their study demonstrated that short-term CPAP improved biventricular function in patients with moderate–severe OSA and cardiometabolic comorbidities, improving left ventricular global longitudinal strain, isovolumetric relaxation time, transmitral E wave amplitude, transmitral E/A ratio, right ventricular diameter, right ventricular wall thickness, right ventricular systolic excursion velocity, and tricuspid annular plane systolic excursion [7]. The article by Dr. Peker and his team provides evidence that improvement in the negative mood symptoms associated with depression was associated with an improvement in the excessive daytime sleepiness reported in coronary artery disease (CAD) patients with sleepy OSA using CPAP treatment, but this association did not exist for cognitive symptoms or for anhedonia and appetite [8]. Using network analysis, Mihaicuta and his coworkers found that neck circumference (>41 cm) is the best qualitative indicator of CPAP treatment response and that it predicts OSA risk [9].

In closing, I would like to thank the authors who contributed to this Special Issue. They covered their respective topics in great depth and clearly explained the issues from clinical perspectives that are important in health care. I also wish to thank reviewers and the editorial staff who contributed to this Special Issue and the many others who conduct research on sleep-related issues and encourage initiatives such as the Sleep Research Society and the American Thoracic Society.

## Data Availability

Not applicable.
